# Synthesis and Structural Studies of New Selenium Derivatives Based on Covalent Functionalization of MWCNTs

**DOI:** 10.3390/ijms24043299

**Published:** 2023-02-07

**Authors:** Sandra Żarska, Rafał Szukiewicz, Sergiu Coseri, Volodymyr Pavlyuk, Dorota Krasowska, Wojciech Ciesielski

**Affiliations:** 1 Institute of Chemistry, Faculty of Science and Technology, Jan Dlugosz University in Czestochowa, 13/15 Armii Krajowej Ave., 42-200 Czestochowa, Poland; 2 Faculty of Physics and Astronomy, Institute of Experimental Physics, University of Wroclaw, 50-204 Wroclaw, Poland; 3 Polyaddition and Photochemistry Department, Petru Poni Institute of Macromolecular Chemistry, 41A Grigore Ghica Voda Alley, 700487 Iasi, Romania; 4 Division of Organic Chemistry, Centre of Molecular and Macromolecular Studies, Polish Academy of Sciences, 112 Sienkiewicza Str., 90-001 Lodz, Poland

**Keywords:** nanomaterials, organoselenophosphorus compounds, covalent functionalization, nanotubes

## Abstract

Modifying the surface of nanomaterials, such as carbon nanotubes, by introducing heteroatoms or larger functional groups into the structure causes a change in chemical properties—manifested in the increase in reactivity as well as a change in conductivity. This paper presents the new selenium derivatives obtained by a covalent functionalization of brominated multi-walled carbon nanotubes (MWCNTs). The synthesis was carried out in mild conditions (3 days at room temperature), and was additionally assisted with ultrasound. After a two-stage purification, the obtained products were identified and characterized by the following methods: scanning and transmission electron microscopy imaging (SEM and TEM), energy dispersive X-ray microanalysis (EDX), X-ray photoelectron spectroscopy (XPS), Raman and nuclear magnetic resonance (NMR), and X-ray diffraction (XRD). In the selenium derivatives of carbon nanotubes, the content of selenium and phosphorus reached 14 and 4.2 wt%, respectively.

## 1. Introduction

The constant search for new and advanced materials is an important characteristic for the development of new technologies. In recent years, much attention has been paid to nanostructured materials with different chemical compositions in the form of nanoparticles, nanowires, or nanotubes. The unwavering interest in their production, properties, and applications is particularly noticeable in the yearly growth of the number of new scientific studies, publications, and patent applications [1,2].

In the early 1990s, the development of research on multi-walled CNTs began. Their production was first developed in the cathode deposit of the carbon arc by the Japanese physicist Iijima [3]. Single-walled carbon nanotubes (SWCNTs) are the most homogeneous and have the fewest network defects; meanwhile, in MWCNT structures, there are various types of defects (structural, doping, interstitial, gaps) that affect their physicochemical properties. Despite the simple chemical composition and configuration of atomic bonds, CNTs show the greatest variety in terms of structure and properties resulting from their structure among all nanomaterials [4,5].

A major problem when working with CNTs is their agglomeration, leading to difficulties in their dispersion when applied to classic organic solvents and a polymer medium [6]. Additionally, nanotubes are insoluble in water due to their hydrophobic nature. Pristine CNTs are weakly chemically reactive. The formation of chemical bonds with surrounding molecules is greatly hampered by the presence of sp^2^ hybridized carbon atoms in the hexagonal nanotube lattices [7]. All these cause great limitations in their use. By modifying the surface properties of CNTs, not only can solubility be improved, but also other physical properties and chemical reactivity can be changed. This is possible in two ways: (1) covalent attachment of the functional groups to nanotube ends or side walls, especially in areas with structural defects [8], and (2) non-covalent supramolecular adsorption (for example with biomolecules) or wrapping of various functional molecules on nanotubes [9,10,11]. 

Functionalization of CNTs through controlled structural changes is crucial for their application capabilities. Adding functional groups or introducing heteroatoms to the structure of nanotubes can increase their dispersion and processing ability [12,13,14]. Additionally, it enables the adjustment of physicochemical properties [15,16] in order to improve mechanical, thermal, and optical composition [17] in a broad spectrum of applications in materials science [18] and biomedical sciences [16,19,20]. Functionalization is also effective in separating the nanotube bundles [21]. Modification strategies for carbon nanotubes differ mainly in the type of medium used, the conditions of their operation, and the presence of additional substances, including catalysts. Nevertheless, in order to thoroughly understand the changes resulting from their functionalization, it is necessary to thoroughly characterize the surface chemistry and structure of CNTs [22,23,24].

Covalent functionalization of carbon nanotubes with sulfur-, selenium-, or phosphorus-containing substituents can be performed both in a single reaction or in multiple steps. In one-step functionalization, bonds are formed between the C atoms on the surface of the CNTs and the reactive center of the substituent (or substituents). The multi-step approach uses at least two reactions: the main one involving the attachment of heteroatoms, such as halogens [14,25,26] or others of the so-called “good” leaving group and a secondary group, which, through a nucleophilic substitution reaction, allows the selective introduction of more complex compounds onto the CNT surface. In the case of the synthesis of MWCNT derivatives containing a phosphorus and sulfur heteroatom, the reactions were based on the nucleophilic substitution of their halogenated (mainly brominated) structures. The modifications that are carried out so far have improved the electrochemical properties in relation to the starting forms, thus making it possible to use them as energy storage materials, including components of lithium-ion cells [27,28,29].

This work presents a synthesis and structural studies of new functional materials based on multi-walled carbon nanotubes modified with organoselenophosphorus substituents. For this purpose, new sodium and lithium *O,O*-dialkyl phosphoroselenoates were synthesized. The next step involved the covalent attachment of bromine to MWCNTs in order to improve their chemical activity. The detailed procedure to obtain brominated multi-walled carbon nanotubes, as well as their structure and properties, were presented in our previous work [30] based on our patent [26]. Finally, covalent functionalization of the brominated MWCNTs with phosphoroselenoates was performed. This kind of material can be used as potential components for lithium-ion cells [31]. Their thermal and electrochemical properties will be discussed in the next paper.

## 2. Results and Discussion

**Figure 1:**
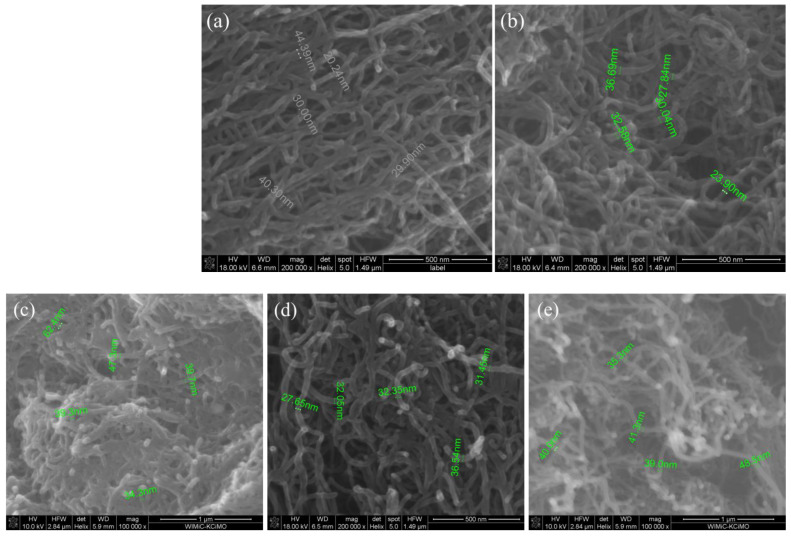
SEM images of MWCNTs: (**a**) pristine, (**b**) brominated and functionalized, (**c**) MWCNT-I, (**d**) MWCNT-II, and (**e**) MWCNT-III.

The SEM microscopic images of the pristine and modified MWCNTs are shown in Figure 1. It can be seen that the performed functionalization influenced the structure of the nanotubes. After the modification, the outer diameters of the nanotubes ranged from 32 to 45 nm. For the MWCNT-I and -III systems, the modifications with phosphoroselenoates resulted in an increase in the nanotube diameters (Figure 1c,e), while for MWCNT-II (Figure 1d), a slight decrease in diameter compared to pristine MWCNTs is observed. This situation may be related to the low degree of functionalization for this sample.

Data obtained from EDX elemental composition analysis (carbon, bromine, oxygen, selenium, and phosphorus) of pristine and MWCNT derivatives are presented in Table 1. A chemical analysis in the micro-areas of the purified products showed that the bromine content decreased after a modification with phosphoroselenoates to 1% for the MWCNT-II sample, whereas for the MWCNT-I sample, the highest content of selenium and phosphorus was observed: 14 and 4.2% by weight, respectively.

**Figure 2:**
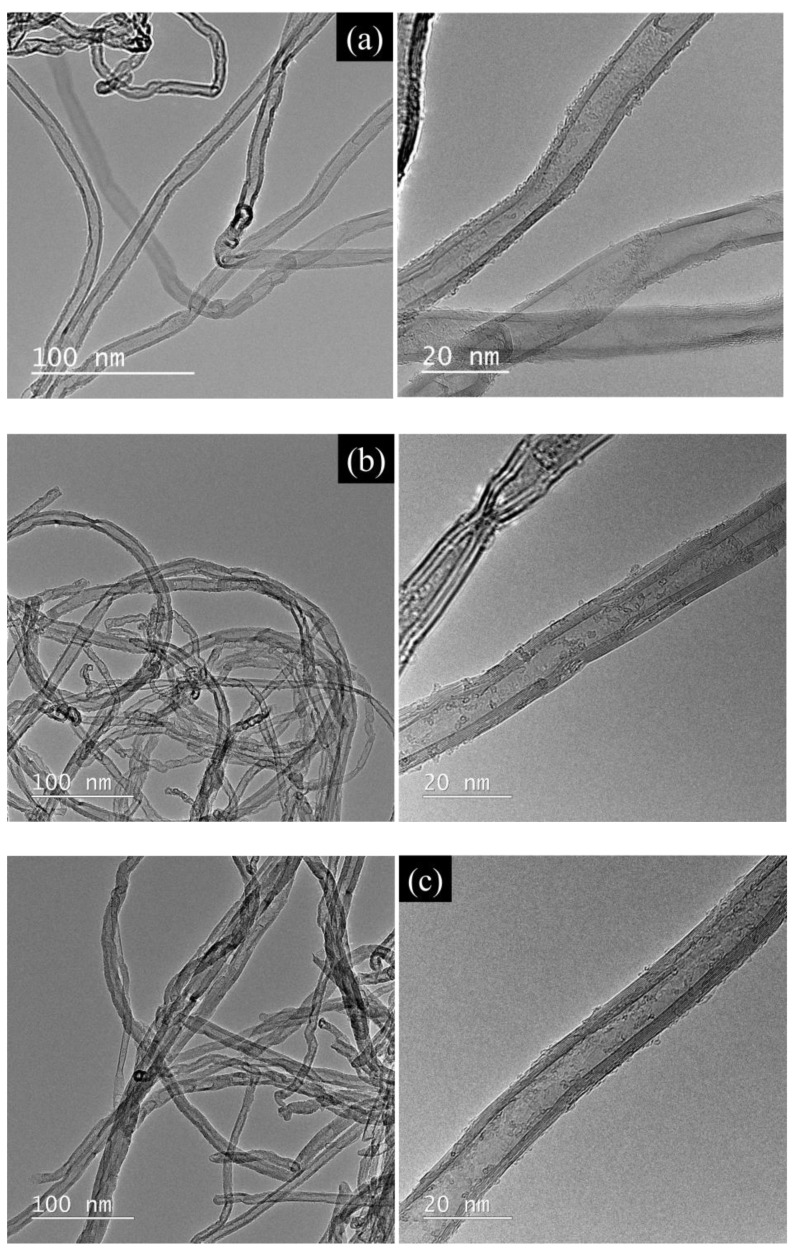

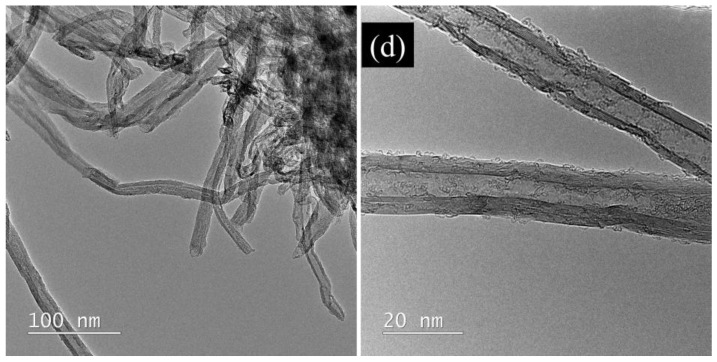
TEM micrographs of the (**a**) initial sample, (**b**) MWCNT-I, (**c**) MWCNT-II, (**d**) MWCNT-III.

The morphology of the internal structure of the multi-walled carbon nanotubes was visualized using transmission electron microscopy (TEM) and high-resolution transmission electron microscopy (HRTEM) (Figure 2). Pictures that were taken using a transmission microscope allow for the estimation of the type and distribution of contaminants that may be present inside or on the surface of the nanotubes. After introducing the functional groups (bromine [30], selenophosphates), nanotubes were deformed (bent at the places where heptagon–pentagon pairs occur), opening at their ends and exfoliating at the inner walls. As a result of the functionalization, physical adsorption of amorphous carbon fragments was observed on the surface of the inner and outer walls. The walls of the derivatives of the nanotubes were more folded compared to the starting material [32].

X-ray photoelectron spectroscopy (XPS) is a qualitative and quantitative technique to measure the elemental composition of a material surface. In the process of deconvolution, it also allows for the identification of the types of bonds of elements present on the examined surface. The total concentrations of atoms in the tested samples were determined on the basis of spectra recorded in a wide range of binding energies (from 0 to 1200 eV, with a measurement step of 0.33 eV, energy of the transition at 100 eV). The spectra of individual elements (C, O, Br, P, Se, Na, Li, C KLL) were also measured with greater accuracy (0.03 eV measurement step, 100 eV energy of the transition). On the basis of the obtained results, the concentration percentage of atoms for the tested samples was determined, and its results are presented in Table 2.

To determine the types of carbon bonds (sp^2^/sp^3^) for the tested samples, the Auger parameter (D parameter) was determined. Based on the differentiated area of Auger excitations of carbon (C KLL approximately 980 eV), by measuring the distance between the minimum and maximum in the differentiated curve, parameter D was determined (Table 2).

The determined D-Auger parameters indicate the presence of both sp^2^ and sp^3^ bonds. In the case of MWCNT-I and MWCNT-II samples, the material behaves like graphite (21 eV), and, for MWCNT-III, like diamond (13 eV) [33]. On the basis of the obtained XPS results shown in Table 2, it can be concluded that the Br atomic concentration in brominated nanotubes was 0.68%. However, for the elements P and Se, the highest atomic concentration was observed for the MWCNT-I sample: 2.4 and 0.5%, respectively. Comparing the starting material with its derivatives, a significant increase in oxygen content is visible, from 1.3 to even 15.9%.

Depending on the applied technique for elemental analysis (EDX) or (XPS), the depth of interaction of the beam with the tested sample layer is different; therefore, the obtained values of the elemental composition of the samples for the above-mentioned methods may differ. The data from EDS measurements presented in Table 1 are related to the sample volume (up to a few µm), while in Table 2, they are XPS data that provide information on the chemical environment of the atoms included in the tested surface layer (the analysis depth of up to nm). 

**Figure 3:**
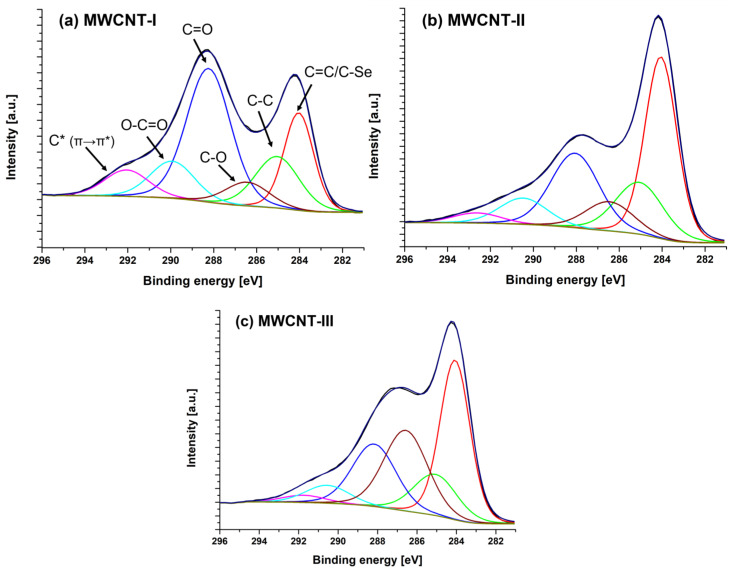
High-resolution C1s regions of (**a**) MWCNT-I, (**b**) MWCNT-II, and (**c**) MWCNT-III samples, indicating the different carbon chemical groups.

Figure 3 presents XPS spectra corresponding to the C1s carbon region for all the analyzed MWCNT samples. Table 3 provides information about carbon content of the functional groups of the MWCNT samples (at%). The area of the C1s of the MWCNT samples functionalized with phosphoroselenoates consisted of carbon groups: sp^2^ C=C/C-Se (284.0 eV), sp^3^ (285.0 eV), C-O (286.4 eV), C=O (288.0 eV), O-C=O (290–291 eV), and π–π* at about 292 eV. C* π→π* is the so-called shake-up satellite that appears at the characteristic energies of the excited states of the element in relation to the state measured by the intensity of zero losses [34]. The binding energy of C-Se is 284.3 eV and can coincide with the binding energy of the sp^2^ carbon occurring at 284 eV; therefore, both bonds are represented by a single peak with an energy of 284 eV.

**Figure 4:**
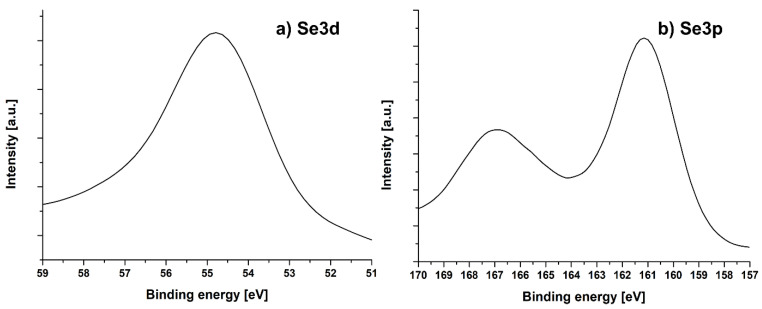
Se3d and Se3p regions for the sample MWCNT-I.

The halogenation with bromine MWCNT carried out in the first stage led to the formation of C-Br bonds and the subsequent stages, including their reactions with selenophosphorus salts to C-Se bonds. In the MWCNT-I sample, the Se3p region consisted of covalently bonded selenium atoms with energies of 54.7 eV for Se3d (Figure 4a) and 167 and 161 eV, respectively, for Se3p_1/2_ and Se3p_3/2_ (Figure 4b) [35].

Analysis of the XPS data of the C1s area confirmed the covalent functionalization of the carbon nanotubes. In the case of bromo-derivative carbon nanotubes, this can be confirmed by reducing the ratio of C-O to C-Br bonds, while in the case of their selenophosphorus derivatives, it is reflected in the change in the share of sp^2^ carbon bonds in favor of sp^3^ bonds (change of the type of hybridization of carbon atoms in the MWCNT network).

**Figure 5:**
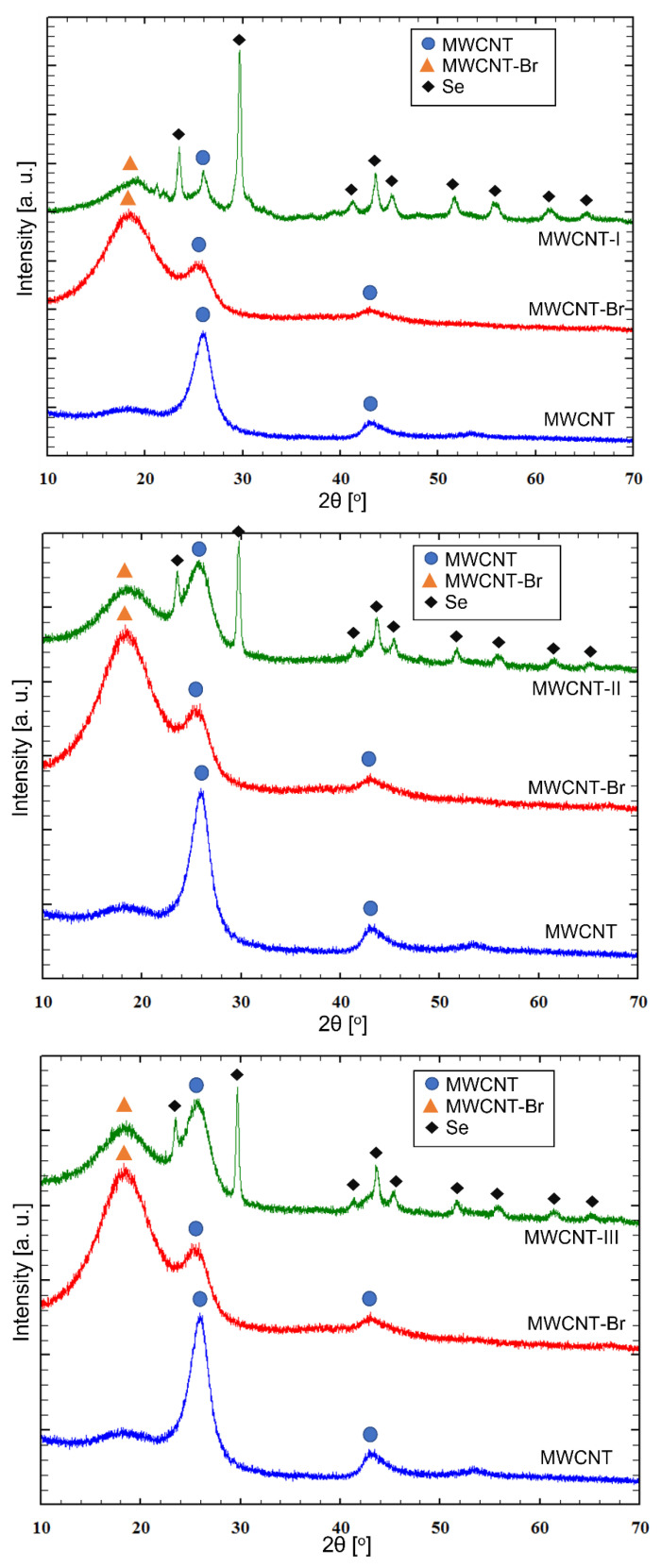
XRD patterns of samples of MWCNTs. The positions of diffraction peaks are marked in blue circles for pristine, orange triangles for brominated MWCNTs, and black rhombuses for selenium.

XRD patterns of pristine, brominated, and selenophosphated nanotubes are shown in Figure 5. A typical diffraction pattern of MWCNTs is characterized by a sharp reflex at 25.7° (2θ) and a broad and weak reflection at 42.9° (2θ) [36,37,38]. The diffraction peaks from pristine nanotubes were observed in the MWCNT-Br diffraction pattern. Additionally, there is a characteristic reflex at 18.3° (2θ) [30]. The shift of the diffraction peaks towards the lower 2θ angles for the bromine modified phase (MWCNT-Br) indicates an increase in the distance between the layers. The diffractograms of phosphoroselenoate-modified nanotubes consisted of the reflections from pristine MWCNT (approximately 26°), brominated (approximately 18°), and confirming the presence of selenium in the samples—e.g., 24, 30, 44, 51° (2θ). A shift of selenium diffraction peaks towards higher 2θ angles is observed. It is worth noting that the intensity of the reflections of the unmodified phase was lower compared to the starting material, and the brominated phase compared to the brominated material.

Crystallographic data calculated on the basis of diffraction spectra of pristine nanotubes and their derivatives, including cell parameters, interlayer distances, and space group, are presented in Table 4. The interlayer distance in MWNCTs estimated from X-ray data was 3479 Å. The increase in interlayer distances, caused by the attachment of bromine atoms or selenophosphorus salt (RO)_2_P(O)SeM, and, thus, the shift of reflections, clearly indicates the possibility of modifying MWCNT. It can be noticed that during bromination and the synthesis of their derivatives, nanotubes swell as a result of the expansion of C =C bonds and interlayer distances, while the space group is unchanged.

**Figure 6:**
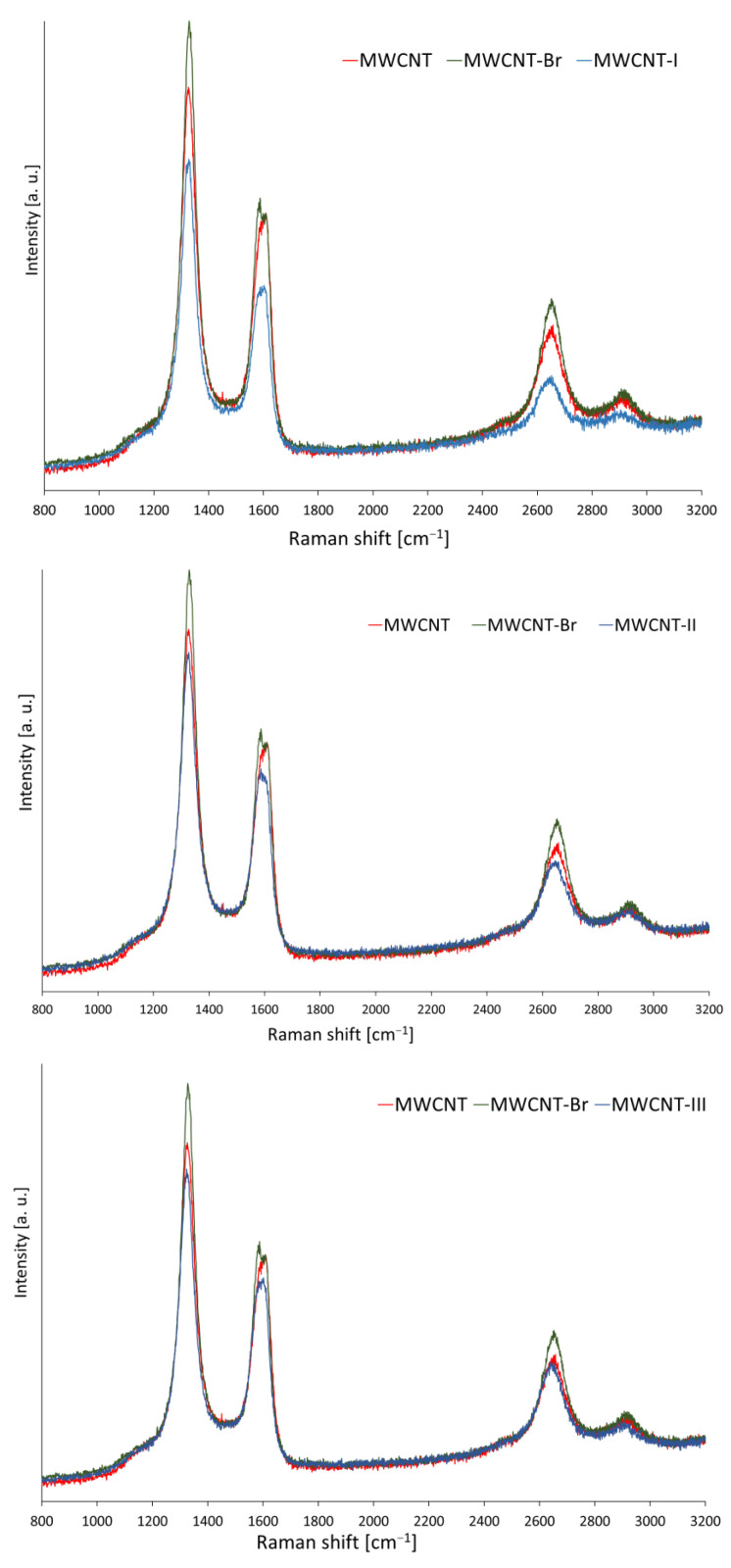
Raman spectra for pristine, brominated MWCNT and functionalized with phosphoroselenoates.

The Raman spectrum of carbon nanotubes provides information on the number of defects or the degree of functionalization [39,40,41]. In the Raman spectra of pristine and functionalized MWCNTs, three main peaks (D, G, and G’) were observed (Figure 6); while the RBM (radial breathing mode, derived from the “breathing movement” of nanotubes) band, which is characteristic only for SWCNT, was not observed. The location of the D-band (approximately 1326 cm^−1^) is related to the disorganization of the structure and, thus, to possible defects in the carbon network of nanotubes; while the G-band (approximately 1595 cm^−1^) is characteristic of the graphite structure and comes from the movements of carbon atoms along and across the CNT axis, its intensity determining the degree of graphitization of the sample. Moreover, the G’ band (D-band overtone, approximately 2646 cm^−1^) is not related to structure disturbances, but it proves the presence of the graphene structure.

Table 5 compares the Raman shift positions and the D-to-G band intensity ratio for the MWCNT before and after the modifications. Slight shifts of the signals from the modified MWCNTs with respect to the frequency of the signals for the starting material were observed. MWCNTs are characterized by a higher D-band intensity than SWCNTs, which may indicate contamination of the sample with amorphous carbon in the case of the starting nanotubes or the presence of covalent bonds between the nanotube and the introduced chemical moiety [42,43]. For all samples, the intensity of the D band is greater than G (Figure 6), which is consistent with the literature [44].

The introduced selenophosphorus and bromine groups did not affect the shape of the Raman spectrum but influenced the intensity of the D, G, and G′ bands. In the case of brominated nanotubes, the intensity of these bands has significantly increased compared to the starting form.

More information about the chemical modification of nanotubes is provided by comparing the intensity quotient (I_D_/I_G_) obtained from the calculations from the fitted Lorentz regions for the D and G bands. It proves the defect content and is a direct measure of the quality of the material [45]. The observed increase in the I_D_/I_G_ value from 1.34 for unmodified MWCNTs to 1.47 for MWCNT-Br indicates a larger structure disorder (related to the change of the hybridization of carbon atoms in the MWCNT network from sp^2^ to sp^3^). The decrease in the I_D_/I_G_ value for the modified nanotubes with phosphoroselenoates suggests a higher structural quality (greater purity) of the samples as a result of the repeated washing of the nanotubes, removing or filling defects and introducing functional groups. The highest value of this ratio was achieved by the nanotubes modified with sodium *O,O*-di-*t*-butyl phosphoroselenoate (MWCNT-I) amounting to 1.43.

The observed shifts of the Raman signals of the D, G, and G′ bands indicate the rearrangement and change of the hybridization type, thus indicating the formation of covalent bonds between the nanotube and the introduced functional groups. Furthermore, the changes in the I_D_/I_G_ ratio show the difference between the starting material and the modified material.

## 3. Materials and Methods

### 3.1. Materials

All reagents were of analytical grade. They were purchased from Sigma-Aldrich (Germany). MWCNTs NC7000 were manufactured by NANOCYL^TM^ (Sambreville, Belgium), which were produced through the catalytic chemical vapor deposition (CCVD). Their average diameter and length were 9.5 nm and 1.5 µm, respectively. They consisted of 90% carbon and <1% metal oxide and had a surface area of 250–300 m^2^g^−1^ [46].

### 3.2. Synthesis of O,O-Dialkyl Phosphoroselenoates

New compounds were synthesized: *O,O*-di-*t*-butyl and *O,O*-bis(2-ethylhexyl) sodium phosphoroselenoates in reactions with alkoxides and *O,O*-di-*n*-butyl lithium phosphoroselenoate with alkali metal hydride, based on the procedures described by Bruzik et al. [47] or by those collected in the review [48]. The structures of the obtained sodium (lithium) *O,O*-dialkyl phosphoroselenoates were confirmed by ^1^H and ^31^P nuclear magnetic resonance spectroscopy.

Compound spectra analysis (*t*-C_4_H_9_O)_2_P(O)SeNa ^1^H (200 MHz, CDCl_3_), (δ, ppm): 1.55 (singlet, 9H, -C(C**H**_3_)_3_). $\delta_{31_{P}}$= 28.4 ppm. ^1^*J*_PSe_ = 754 Hz (81 MHz, CDCl_3_). 

Compound spectra analysis (*n*-C_4_H_9_O)_2_P(O)SeLi: ^1^H (500 MHz, CDCl_3_), (δ, ppm), 0.87 (triplet, *J*_PH_ = 15 Hz, 3H, -CH_2_-C**H**_3_); 1.29–1.35 (sextet, *J*_PH_ = 40 Hz, *J*_HH_ = 10 Hz, 2H, -CH_2_-C**H**_2_-CH_3_); 1.51–1.57 (sextet, *J*_PH_ = 35 Hz, *J*_HH_ = 5 Hz, 2H, -CH_2_-C**H**_2_-CH_2_-); 3.78–3.88 (multiplet, 2H, O-C**H**_2_-CH_2_-). $\delta_{31_{P}}$= 52.2 ppm; ^1^*J*_PSe_ = 776.5 Hz (202.5 MHz, CDCl_3_). 

Compound spectra analysis (C_8_H_17_O)_2_P(O)SeNa: ^1^H (200 MHz, CDCl_3_), (δ, ppm): 0.86 (triplet, 6H, -(CH_2_-C**H**_3_)_2_); 1.40–1.23 (multiplet, 8H, -(C**H**_2_)_4_)-); 1.60–1.51 (multiplet, 1H, -CH_2_-C**H**(-CH_2_)-CH_2_-); 4.04–3.89 (quartet, 2H, O-C**H**_2_-CH-). $\delta_{31_{P}}$= 62.7 ppm. ^1^*J*_PSe_ = 886 Hz (81 MHz, CDCl_3_).

### 3.3. Functionalization of MWCNT-Br with O,O-Dialkyl Phosphoroselenoates

**Figure 7:**
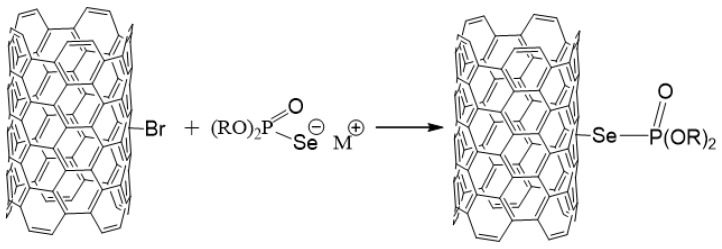
Schematic representation of the MWCNT functionalization procedure. R = *t*-C_4_H_9_; *n*-C_4_H_9_; C_8_H_17_; M = Na, Li.

In order to increase the chemical reactivity of the MWCNT, bromine atoms were introduced into their structure [30]. The bromination process was carried out as described in the European patent no. P.409662 [26]. The brominated MWCNT (MWCNT-Br) was then modified with sodium salts or lithium salt of organophosphorus acid in a mass ratio of 1:3 in dry tetrahydrofuran in a round-bottom flask, the content of which was stirred with a magnetic stirrer for 3 days in an argon atmosphere at room temperature [31]. After this time, the system was subjected to ultrasound (Polsonic ultrasonic bath, 320 W, 40 kHz) for 30 min in order to increase the efficiency and effectiveness of the reaction. The reaction scheme for the preparation of selenophosphorus MWCNT derivatives is presented in Figure 7.

After this time, the crude products were subjected to a two-step purification that consists of washing successively with deionized water and methanol. After each step, the samples were centrifuged and dried for 12 h at 80°C. The resulting compounds with the following summary formulas (*t*-C_4_H_9_O)_2_P(O)SeNa/MWCNT; (*n*-C_4_H_9_O)_2_P(O)SeLi/MWCNT; (C_8_H_17_O)_2_P(O)SeNa/MWCNT were marked in this work as MWCNT-I, MWCNT-II, MWCNT-III, respectively.

### 3.4. Characterization Methods

#### 3.4.1. Nuclear Magnetic Resonance Spectroscopy (NMR)

The composition of the obtained sodium and lithium *O,O*-dialkyl selenophosphates was determined by NMR measurements. ^1^H and ^31^P NMR spectra were recorded on an Ultrashield Avance II spectrometer (Bruker Corporation, Billerica, MA, USA) at 200 and 500 MHz. Deuterated chloroform (CDCl_3_) was used for the measurements.

#### 3.4.2. Scanning Electron Microscopy (SEM)

The morphology of the pristine and modified MWCNT surfaces was examined using a Nova Nano SEM 200 high-resolution scanning electron microscope (FEI Europe Company, Eindhoven, The Netherlands) with a field emission gun (FEG-SCHOTTKY emitter) with a resolution of up to 500 nm and a magnification of 70–200,000. The samples in the powder form were exposed to the beams of the accelerating voltage of 10 kV and 18 kV.

#### 3.4.3. Energy Dispersive X-ray Microanalysis (EDX) Spectroscopy

A Tescan Vega 3 SBU scanning electron microscope (Tescan, Brno, Czech Republic) with a EDX analyzer (Oxford Instruments, Aztec ONE system, High Wycombe, UK) was used for the spectroscopic analysis in the micro-areas by the X-ray energy dispersion method.

#### 3.4.4. Transmission Electron Microscopy (TEM)

Samples were characterized using a FEI Titan^3^ G2 (S) TEM 60–300 dual correction transmission electron microscope (Thermo Fisher Scientific, Hillsboro, OR, USA) equipped with an X-FEG emitter, beam monochromator, and image and beam corrector. The tests were carried out at an accelerating voltage of 80 kV. A small amount of powder was applied to a copper TEM mesh covered with a lacey carbon film. The samples were observed without the use of additional solvents.

#### 3.4.5. X-ray Photoelectron Spectroscopy (XPS)

For surface chemical composition analysis X-ray photoelectron spectroscopy (XPS) has been applied. All measurements have been performed using an XPS/AES system EA10 (Leybold-Heraeus GmbH, Cologne, Germany). The non-monochromatized X-ray Mg K*α* excitation source was used. The overall resolution of the spectrometer during the measurements was 0.96 eV as a full width of half maximum (FWHM) of the Ag3d_5/2_ line. The base pressure during measurements was in a range of 10^−9^ mbar. After subtraction of the Shirley-type background, the core-level spectra were decomposed into main components with mixed Gaussian–Lorentzian lines (70% G + 30% L for majority of photo-peaks) by a non-linear least squares curve-fitting procedure, using CasaXPS software. The atomic concentration was determined on the basis of XPS spectra analysis, taking into account the presence of individual elements C, O, Br, P, Se, Na, and Li. The D-Auger parameter was determined for all samples, and all the spectra obtained were calibrated for sp^2^ binding at 284 eV [33].

#### 3.4.6. X-ray Diffraction (XRD)

The effectiveness of the modification of the MWCNTs was assessed by the X-ray diffraction method. A Rigaku MiniFlex 600 powder diffractometer (Rigaku, Tokyo, Japan) was used to determine the diffraction spectra of the materials. The X-ray source was a K*α* copper anode lamp, a nickel optical filter was used, and an operating voltage of 45 kV and an operating current of 15 mA were set under normal measurement conditions. The step scan measurement mode was used with a scattering angle of 2θ in the angular range from 10 to 100° with a 1 s step size equal to 0.02° (2θ). The structures were refined using the Rietveld method using the procedures of the FullProf software package (June 2015 version) [49].

#### 3.4.7. Raman Spectroscopy

Raman spectra of MWCNT and their derivatives were recorded in a Raman spectrometer (Renishaw, Wotton-under-Edge, UK) in the spectral range 100–3200 cm^−1^ using a He-Ne laser with a wavelength of 633 nm and power of 17 mW, with the time of exposure to the sample 10 s. The spectra were generated at 5% laser power. Leica confocal microscope resolution was below 2 µm, diffraction grating 1800 lines/mm, 50× magnification.

## 4. Conclusions

The proposed methodology for preparing new, functional nanomaterials uses, inter alia, the electrophilic nature of carbon atoms in halogenated CNTs, which enables the nucleophilic substitution reaction. The formation of covalent bonds between the nanotube and the introduced chemical groups was indicated by a change in the order and the type of hybridization of the carbon atoms. The preparation of the selenium derivatives of MWCNTs was confirmed by many methods: microscopies SEM and TEM; spectroscopic NMR, EDX, XPS, and Raman; as well as by X-ray diffraction. Based on the analyses carried out, it can be concluded that the best degree of functionalization was achieved for MWCNT modified with sodium *O,O*-di-*t*-butyl phosphoroselenoate (the highest I_D_/I_G_ value and atomic concentration of elements).

Carbon nanotubes, due to their unique properties, such as a large specific surface area wettable by the electrolyte; very good electrical conductivity; and high chemical, mechanical, and electrochemical stability, are potentially a good material for electrodes, e.g., in lithium-ion batteries. Therefore, in the next paper, electrochemical and thermal studies of the obtained functionalized MWCNTs and the possibility of their use as energy storage materials will be carried out. The ability to store energy will be tested in three-electrode cells as well as coin cells by performing the following analyses: chronopotentiometry and cyclic voltammetry. In addition, their corrosion resistance will be assessed.

## 5. Patents

The process of preparing the brominated multi-walled carbon nanotubes (MWCNT) containing bromine atoms and their purification method. Drabowicz, J.; Ciesielski, W.; Kulawik, D. Pat. EP3002253, 19 February 2015. 

Functionalized multi-wall carbon nanotubes, method of their production and their application. Ciesielski, W.; Kulawik, D.; Żarska, S.; Folentarska, A.; Drabowicz, J. Pat. 240569, 31 January 2019.

## Figures and Tables

**Table 1:** The composition of chemical elements in the analyzed samples by the EDX method (wt%).

General Chemical Formula	Sample	C	Br	O	Se	P
-	MWCNT	90.5	-	9.5	-	-
-	MWCNT–Br	90.2	7.3	2.6	-	-
(*t*-C_4_H_9_O)_2_P(O)SeNa/MWCNT	MWCNT-I	60.9	3.2	14.0	14.0	4.2
(*n*-C_4_H_9_O)_2_P(O)SeLi/MWCNT	MWCNT-II	96.5	1.0	1.8	0.6	0.2
(C_8_H_17_O)_2_P(O)SeNa/MWCNT	MWCNT-III	82.8	7.1	7.8	1.7	0.5

**Table 2:** Atomic concentration (at%) and D parameter (eV) from XPS survey spectra.

Samples	C	O	Br	P	Se	Na	Li	D-Parameter
MWCNT	98.58	1.32	-	-	-	-	-	21.8
MWCNT–Br	95.36	3.95	0.68	-	-	-	-	20.0
MWCNT-I	79.10	15.90	0	2.40	0.49	2.11	-	18.0
MWCNT-II	92.70	6.80	0.16	0.25	0,06	-	0.03	19.3
MWCNT-III	90.42	8.64	0	0.52	0.1	0.32	-	16.3

**Table 3:** Comparison of carbon species concentration data (at%) from C1s regions after the deconvolution analysis.

Sample	C1s [eV]					
	C (sp^2^) C=C/C-Se 284.0	C (sp^3^) C-C 285.0	C-O 286.4	C=O 288.0	O-C=O 290–291	π–π*
MWCNT	64.2	15.5	4.4	4.8	7.8	2.4
MWCNT-I	17.33	18.58	4.22	35.61	13.67	10.59
MWCNT-II	38.54	16.59	9.35	23.85	8.4	3.26
MWCNT-III	34.34	12.91	25.29	19.59	5.62	2.26

**Table 4:** XRD parameters for selected MWCNTs samples.

Samples	Lattice Parameters [Å]	Interlayer Distances [Å]	Space Group
MWCNT	a = 2.523 c = 6.958	3.479	P63/mmc
MWCNT–Br	a = 2.652 c = 9.258	4.629	P6_3_/mmc
MWCNT-I	a = 2.624 c = 9.233	4.616	P6_3_/mmc
MWCNT-II	a = 2.644 c = 9.245	4.623	P6_3_/mmc
MWCNT-III	a = 2.632 c = 9.238	4.619	P6_3_/mmc

**Table 5:** Raman shifts for MWCNT before and after the functionalization.

Samples	Type of Vibration [cm^−1^]			Intensity [a.u.]			I_D_/I_G_
	D	G	G′	D	G	G′	
MWCNT	1327	1600	2651	10,262	7658	5049	1.34
MWCNT–Br	1329	1595	2650	11,568	7863	5660	1.47
MWCNT-I	1326	1594	2643	8681	6054	3961	1.43
MWCNT-II	1325	1592	2642	9640	6958	4687	1.39
MWCNT-III	1325	1594	2642	9543	7088	4901	1.35

## Data Availability

Data are contained within the article.
